# Divergent signalling pathways regulate lipopolysaccharide-induced eRNA expression in human monocytic THP1 cells

**DOI:** 10.1016/j.febslet.2014.12.026

**Published:** 2015-01-30

**Authors:** James A. Heward, Benoit T. Roux, Mark A. Lindsay

**Affiliations:** Department of Pharmacy and Pharmacology, University of Bath, Bath BA2 7AY, United Kingdom

**Keywords:** Enhancer RNA, Long non-coding RNA, NF-κB, Lipopolysaccharide, Monocyte, ERK and p38

## Abstract

Recent studies have indicated that non-coding RNAs transcribed from enhancer regions are important regulators of enhancer function and gene expression. In this report, we have characterised the expression of six enhancer RNAs (eRNAs) induced in human monocytic THP1 cells following activation of the innate immune response by lipopolysaccharide (LPS). Specifically, we have demonstrated that LPS-induced expression of individual eRNAs is mediated through divergent intracellular signalling pathways that includes NF-κB and the mitogen activated protein kinases, extracellular regulated kinase-1/2 and p38.

## Highlights

• eRNAs are expressed from enhancers and have been shown to regulate gene expression.
• Expression of eRNAs is widespread upon activation of the innate immune response.
• We show that the NF-κB signalling pathway regulates LPS-induced eRNAs.
• Expression of individual eRNAs is also dependent on ERK-1/2 and p38.

## 1 Introduction

Cells of the innate immune response, including circulating monocytes, prevent infection through the rapid removal of invading pathogens [1]. Monocytes are able to detect micro-organisms by a variety of mechanisms including the use of membrane associated pattern recognition receptors (PRR) that bind to conserved components upon the pathogens. The best described is the Toll-like receptor (TLR) family and in particular TLR4, which is activated by lipopolysaccharide (LPS), a component of gram-negative bacteria cell walls. TLR4 activation triggers intracellular signalling cascades, most notably those that lead to activation of the transcription factor, nuclear factor kappa-light-chain-enhancer of activated B cells (NF-κB). In addition, LPS stimulation has been shown to stimulate multiple mitogen activated protein kinase (MAPK) signalling pathways including extracellular signal-regulated kinases (ERK1/2), c-Jun N-terminal kinases (JNK1/2) and p38 MAPK [2]. This ultimately results in the activation of additional transcription factors involved in the antimicrobial response including activator protein (AP-1) and cAMP-responsive-element-binding protein 1 (CREB1) [3].

One of the mechanisms by which the expression of individual genes can be potentially regulated by transcription factors is through interaction with enhancers, regulatory regions of the genome distally located from protein coding genes [4]. This is thought to result in the looping of the chromatin structure which brings the enhancer region into close proximity with the promoters of regulated genes [5,6]. Interestingly, recent studies have indicated that the action of enhancers is dependent upon the transcription of non-coding RNAs, entitled enhancer RNAs (eRNAs) [7]. These are involved both in the establishment of enhancer sites and in the subsequent looping and regulation of gene expression in *cis* [8–13].

In previous reports, ourselves [13] and other investigators [14] have identified widespread induction of eRNAs following activation of the innate immune response. In this report, we have characterised the expression of a range of eRNAs that are induced in response to LPS stimulation of monocytic THP-1 cells. Since little is known about the signalling pathways that regulate the production of these immune-related eRNAs, we have examined the role of NF-κB and the MAPK intracellular pathways and shown that the expression of eRNAs is regulated by multiple divergent mechanisms.

## 2 Materials and methods

### 2.1 Culture of human monocytic THP-1 cells

Monocytic THP-1 cells were cultured in RPMI 1640, supplemented with 10% (v/v) FBS, l-glutamine (2 mM), 1% (v/v) Pen-Strep and 0.1% (v/v) β-mercaptoethanol (Invitrogen Gibco) and cultured in a 37 °C, 5% (v/v) CO_2_ humidified incubator.

### 2.2 Stimulation and drug treatment of monocytic THP-1 cells

Monocytic THP-1 cells were seeded at 5 × 10^5^ cells/well in 500 μl in 24 well plates. Cells were treated with media or 1 μg/ml LPS (*Escherichia* *coli* 055:B5, Sigma Aldrich) for 0–24 h (*n* = 3/group/time-point). For the TLR agonists experiment, cells were treated with PAM_3_CSK4 (100 ng/ml), HKLM (10^8^ cells/ml), poly(I:C) (10 mg/ml), poly(I:C) LMW (10 mg/ml), Flagellin (100 ng/ml), FSL-1 (100 ng/ml), Imiquimod (5 μg/ml), ssRNA40 (1 μg/ml) and ODN2006 (2 μM) (all from InvivoGen). In addition, THP-1 cells were also treated with buffer (control), LPS and IL-1β (10 ng/ml, recombinant, *E. coli*, Sigma Aldrich). For drug treatments, cells were incubated with DMSO (vehicle control) or 0.1–10 μM of TPCA-1 (IκB kinase-2 inhibitor, Sigma–Aldrich), PD0325901 (MEK inhibitor, Tocris) and SCIO 469 (p38 inhibitor, Tocris) for 30 min, prior to stimulation with LPS for 4 h.

### 2.3 RNA isolation and cDNA synthesis

Total RNA was extracted using the Qiagen RNeasy kit which included an on-column DNase treatment. cDNA was synthesised using the High Capacity cDNA Reverse Transcription kit (Applied Biosystems).

### 2.4 qRT-PCR quantification of RNA expression

Expression of eRNAs, mRNAs and 18S RNA was determined by qRT-PCR using the SYBR® Green PCR mix (Applied Biosystems). The separate well, 2^−^^(ΔΔ^*^Ct^*^)^ method was used to determine relative-quantities of individual mRNAs and eRNAs which were normalised to 18S RNA. Primers used are listed in Table 1.

### 2.5 Nuclear-cytoplasm RNA fractionation

Monocytic THP-1 cells were stimulated with LPS for 2 h or 6 h. The cells were centrifuged and then split into two equal fractions. Total RNA was extracted from one fraction using the normal Qiagen RNeasy protocol whilst the other fraction was treated with RLN buffer on ice for 5 min, in order to lyse the plasma membrane whilst leaving the nuclei intact. The nuclei were then isolated by centrifugation at 300×g in a pre-chilled centrifuge. RNA was extracted from the nuclear and cytoplasm fractions using the normal Qiagen RNeasy protocol. In order to quantify gene expression within the different fractions by qRT-PCR, the 18S values from the total RNA fraction were used to normalise the data.

### 2.6 Transfection of monocytic THP-1 cells with anti-p65 siRNAs

Monocytic THP-1 cells were seeded at 2.5 × 10^5^ cells/well in 24 well plates, in 100 μl of complete growth medium. Transfection mixes were prepared using 95 μl of serum-free growth medium, 5 μl of HiPerFect (QIAGEN) plus siRNAs to give a final concentration of 30 nM. Cells were subsequently incubated for 6 h, diluted with 400 μl of complete growth medium, incubated for a further 42 h and then stimulated with LPS for 2 h. siRNA sequences are listed in Table 1.

### 2.7 Detection of p65 and MAP kinases using Western blotting

Monocytic THP-1 cells were seeded at 1.5 × 10^6^ cell/well in 12 well plates and transfected with siRNAs (see above). Cells were lysed in buffer containing 50 mM Tris–HCl, 150 mM NaCl, 1% (v:v) Nonidet P40, 1 mM sodium vanadate, 1 mM sodium molybate, 10 mM sodium fluoride, 40 μg/ml PMSF, 0.7 μg/ml pepstatin A, 10 μg/ml aprotinin, 10 μg/ml leupeptin and 10 μg/ml soybean trypsin inhibitor. Lysed samples were subsequently diluted in sample buffer containing 5% β-mercaptoethanol, boiled and then separated by electrophoresis on a 10% SDS–PAGE gel. Proteins were transferred to a nitrocellulose membrane and probed with rabbit anti-p65 (NF-κB p65, C-20, Santa Cruz), rabbit anti-pERK-1/2, p-p38, ERK-1/2 and p38 (Phospho-MAPK Family Antibody and MAPK Family Antibody Sampler kit, Cell Signaling) or rabbit anti-GAPDH (D16H11, Cell Signaling Technology). The membranes were subsequently incubated with HRP-conjugated goat anti-rabbit secondary antibody (DAKO) and bands visualised using an EZ-ECL chemiluminescence detection kit (Geneflow, Staffordshire, UK) and ImageQuant developer (GE healthcare, Buckingamshire, UK).

### 2.8 Chromatin Immunoprecipitation

Chromatin immunoprecipitation (ChIP) was performed according to the manufacturer’s guidelines (Active Motif; 53040). In brief, 3 × 10^7^ THP1 cells were stimulated or not with LPS (1 μg/ml) for 60 min. Whole cells were cross-linked with a 1% formaldehyde solution for 15 min at room temperature. Cells were sonicated (Branson Sonifier 250) for 2 cycles (output: 1, duty cycles: 20%, time: 30 s on 30 s off). DNA concentrations were quantified, and 10 μg of chromatin DNA was used for each ChIP reaction. ChIP assays were performed with 4 μg of antibody (NFκB p65, C-20, Santa Cruz) and incubated overnight at 4 °C, precipitated with agarose beads (supplied) and washed. Bead-bound DNA was reverse cross-linked and purified with DNA Purification columns (supplied). Samples were then analysed by qPCR using the probes listed in Table 1.

## 3 Results

### 3.1 eRNAs are differentially expressed following LPS stimulation

**Fig. 1:**
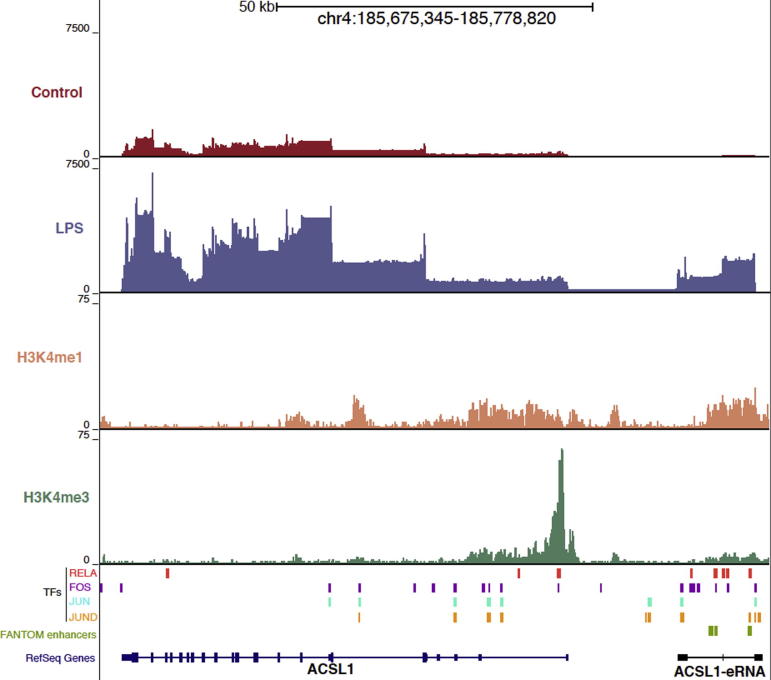
Example of immune-regulated eRNA in monocytic THP-1 cells. eRNAs genes are demarcated by high H3K4me1 and expressed co-ordinately with neighbouring genes. ACSL1 is an example of a protein coding gene with an upstream eRNA gene, both of which are upregulated upon LPS stimulation. RNA-seq data from control (red) and LPS stimulated (blue) monocytes are displayed in the top two tracks. The middle two panels display ChIP-seq data from human monocytes indicating the methylation status of H3K4, with H3K4me1 (enhancer mark) displayed in orange and H3K4me3 (promoter mark) in green. The bottom panel displays transcription factor binding sites identified in the ENCODE project [23], transcribed enhancers identified in the FANTOM project [16] and gene annotations for *ACSL1* from RefSeq (blue) and *ACSL1-eRNA* (black; [13]). Figure produced using the UCSC genome browser.

In a previous publication, we used RNA-seq to interrogate the transcriptome of control and LPS stimulated human monocytes and identified 76 multi-exonic, mono-directionally transcribed eRNAs that were regulated by LPS plus 35 bi-directionally transcribed eRNAs [13]. eRNAs were defined as having a high H3K4me1/H3K4me3 ratio in line with previous reports [15] and annotated according to the nearest expressed protein coding gene e.g. *IL1β-eRNA*. The LPS-regulated eRNAs displayed co-ordinated expression with neighbouring genes, the majority of which were associated with the inflammatory response. An example of an LPS-induced eRNA neighbouring and co-expressed with the gene *long-chain acyl-CoA synthetase* (*ACSL) 1*, is shown in Fig. 1. ACSL1 esterifies long chain fatty acids, a preliminary step in the biogenesis of lipid mediators including the pro-inflammatory prostaglandin E_2_. *ACSL1-eRNA,* ∼10 kb upstream of *ACSL1*, overlapped with the binding sites of multiple transcription factors shown previously to regulate the inflammatory response, including sub-units of NF-κB (RelA/p65) and AP-1 (c-fos, c-jun) [2]. In addition, *ACSL1-eRNA* overlapped with enhancer regions recently classified by the FANTOM project [16]. The overlap between transcription factor binding sites and the enhancer regions characterised by the FANTOM project was also observed with five other eRNAs; *SLC30A4-eRNA*, *SOCS3-eRNA*, *AZIN1-eRNA*, *TNFSF8-eRNA* and *MARCKS-eRNA* (Table 2).

### 3.2 eRNA expression rapidly peaks following LPS stimulation of THP-1 monocytic cells

**Fig. 2:**
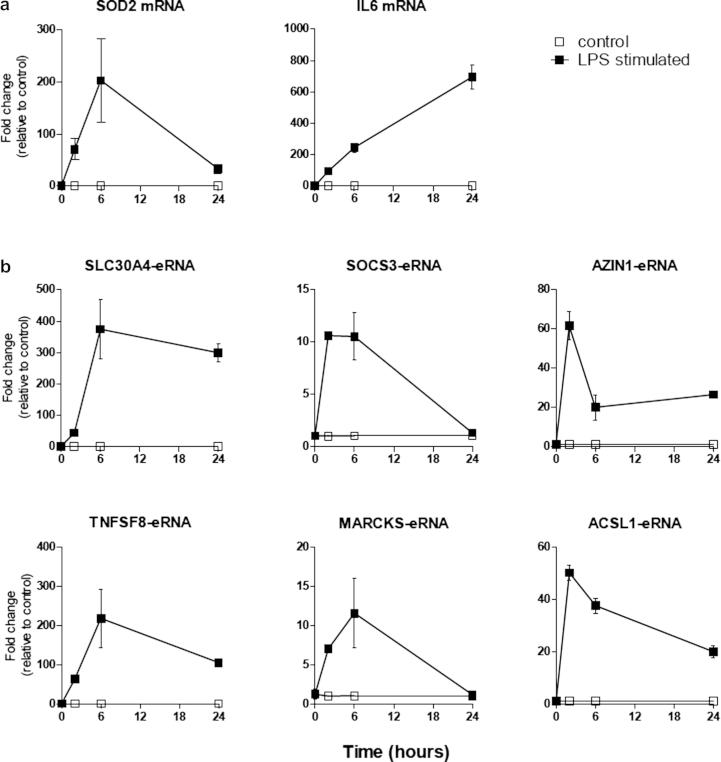
Time course of LPS-induced mRNA and eRNA expression. Human monocytic THP-1 cells were exposed to either buffer or 1 μg/ml LPS for the indicated period of time prior to quantification of (a) *IL6* and *SOD2* mRNA and (b) *SLC30A4-eRNA*, *SOCS3-eRNA*, *AZIN1-eRNA*, *TNFSF8-eRNA*, *MARCKS-eRNA* and A*CSL1-eRNA* by qRT-PCR. Data is the mean ± S.E.M. of 3 independent experiments.

To begin to characterise eRNA expression, we initially examined the time course of LPS-induced expression of two key inflammatory genes, *SOD2* and *IL6* [17–19] and the six immune-related eRNAs, *SLC30A4-eRNA*, *SOC3-eRNA*, *AZIN1-eRNA*, *TNFSF8-eRNA*, *MARCKS-eRNA* and *ACSL1-eRNA*. As expected, we observed a rapid induction of *SOD2* that peaked at 6 h and a steady increase in *IL6* expression throughout the 24 h period (Fig. 2a). All of the eRNAs demonstrated rapid induction with expression peaking between 2 h and 6 h (Fig. 2b).

### 3.3 eRNA expression is induced by TLR4 and TLR6/2 receptor activation

**Fig. 3:**
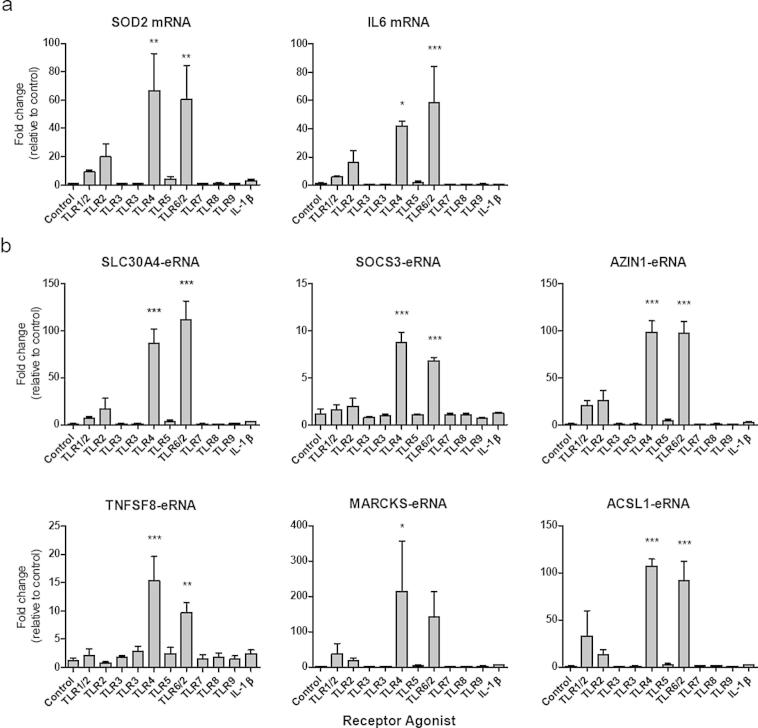
Profile of mRNA and eRNA expression is response to exposure to TLR agonists and IL-1β. Human monocytic THP-1 cells were exposed to buffer, a range of TLR agonists or IL1β for 2 h. TLR agonists included the synthetic bacterial lipoprotein (Pam3CSK4 acting via TLR-1/2), a heat-killed preparation of the gram-positive bacterium Listeria monocytogene (HKLM acting via TLR-2), synthetic mimics of double stranded RNA (polyIC and polyIC LMW acting via TLR-3), the bacterial flagellin protein (acting via TLR-5), a synthetic lipoprotein derived from Mycoplasma salivarium (FSL-1 acting via TLR-2/6), an imidazoquinoline amine analog to guanosine (Imiquimod acting via TLR-7), A GU-rich single stranded RNA (ssRNA40 acting via TLR-8) and a CpG containing oligonucleotide (ODN2006 acting via TLR-9). Expression of (a) *IL6* and *SOD2* mRNA and (b) *SLC30A4-eRNA*, *SOCS3-eRNA*, *AZIN1-eRNA*, *TNFSF8-eRNA*, *MARCKS-eRNA* and *ACSL1-eRNA* was quantified by qRT-PCR. Data is the mean ± S.E.M. of 3 independent experiments. Statistical significance was determined using a one way ANOVA with a Dunnett’s post-test, where ^∗^*P* < 0.05, ^∗∗^*P* < 0.01 and ^∗∗∗^*P* < 0.001.

We next determined the profile of expression following activation of other members of the TLR family (TLR1-9), as well as the inflammatory cytokine IL-1β. Both the inflammatory markers and the eRNAs displayed a similar expression profile, with significant expression only seen in response to LPS (TLR4 agonist) and FSL-1, which acts through the TLR6/2 heterodimer (Fig. 3). Although this needs to be investigated further, it is likely that the latter response is mediated predominantly via TLR6, since the TLR2 agonist (HKLM) had no significant action (Fig. 3).

### 3.4 LPS-induced eRNA expression is predominantly restricted to the nucleus

**Fig. 4:**
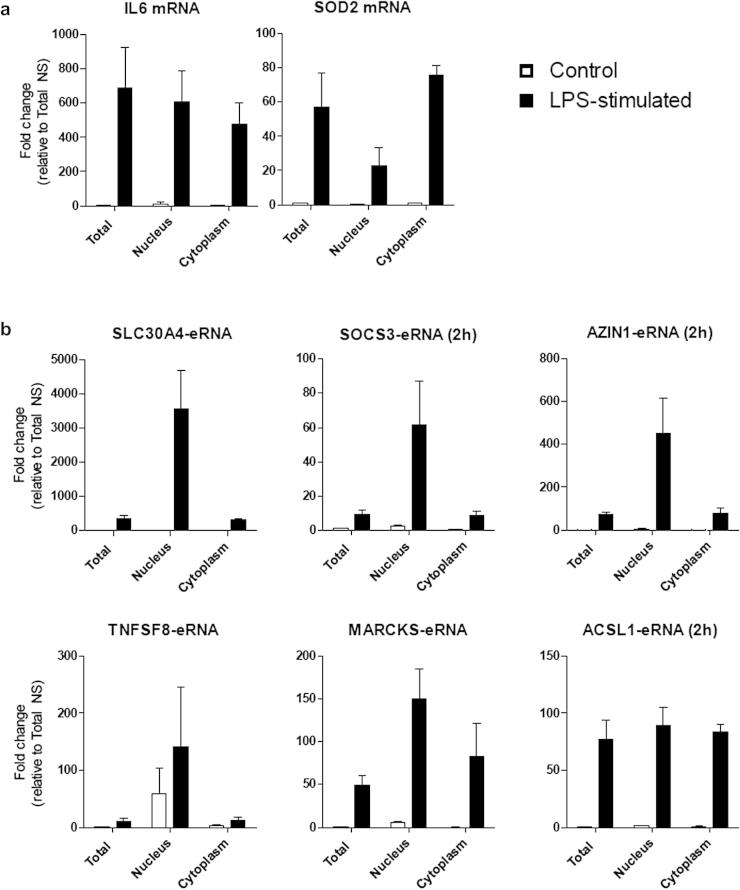
Subcellular distribution of mRNA and eRNA expression. Human monocytic THP-1 cells were stimulated with LPS for 2 h or 6 h prior to RNA extraction from either total cells or the nuclear and cytoplasm fractions. qRT-PCR was then used to analyse the expression and distribution, relative to a total cell fraction, of (a) *IL6* and *SOD2* mRNA and (b) *SLC30A4-eRNA, SOCS3-eRNA, AZIN1-eRNA, TNFSF8-eRNA, MARCKS-eRNA* and *ACSL1-eRNA* by qRT-PCR. Data is the mean ± S.E.M. of 3 independent experiments.

We next sought to determine whether the expression of LPS-induced eRNAs was restricted to the nucleus, consistent with their purported role as regulators of transcription [8,20,21]. To this end, control and LPS stimulated monocytic THP-1 cells were subjected to subcellular fractionation and RNA expression relative to whole cells was examined by qRT-PCR. Successful subcellular fractionation was confirmed following the observation that the nuclear located long non-coding RNA *MALAT1* (metastasis associated lung adenocarcinoma transcript 1) [22] and mitochondrial mRNA *MT-CYB* (mitochondrially encoded Cytochrome B) was restricted to the nuclear and cytoplasmic fractions, respectively (data not shown see Ref. [13]). As supported by the time course data (Fig. 2a), *IL6* expression was found to be equally distributed between the cytoplasm and nucleus, implying on-going transcription whilst *SOD2* was predominant located in the cytoplasm (Fig. 4a). In the case of the eRNAs, 4 (*SCL30A4-eRNA*, *SOCS3-eRNA*, *AZIN1-eRNA* and *TNFSF8-eRNA*) demonstrated strong nuclear enrichment whilst *MARCKS-eRNA* and *ACSL1-eRNA* were expressed in both fractions (Fig. 4b).

### 3.5 LPS-induced eRNA expression is regulated by the NF-κB signalling pathway

Following the activation of TLR4, a number of signalling pathways including those that act through IKK2 (upstream activator of NF-κB) and members of the MAP kinase families are thought to regulate inflammatory gene expression via activation of transcription factors [2]. We therefore sought to determine whether these signalling pathways might also regulate LPS-induced eRNA expression through the use of pharmacological inhibitors.

**Fig. 5:**
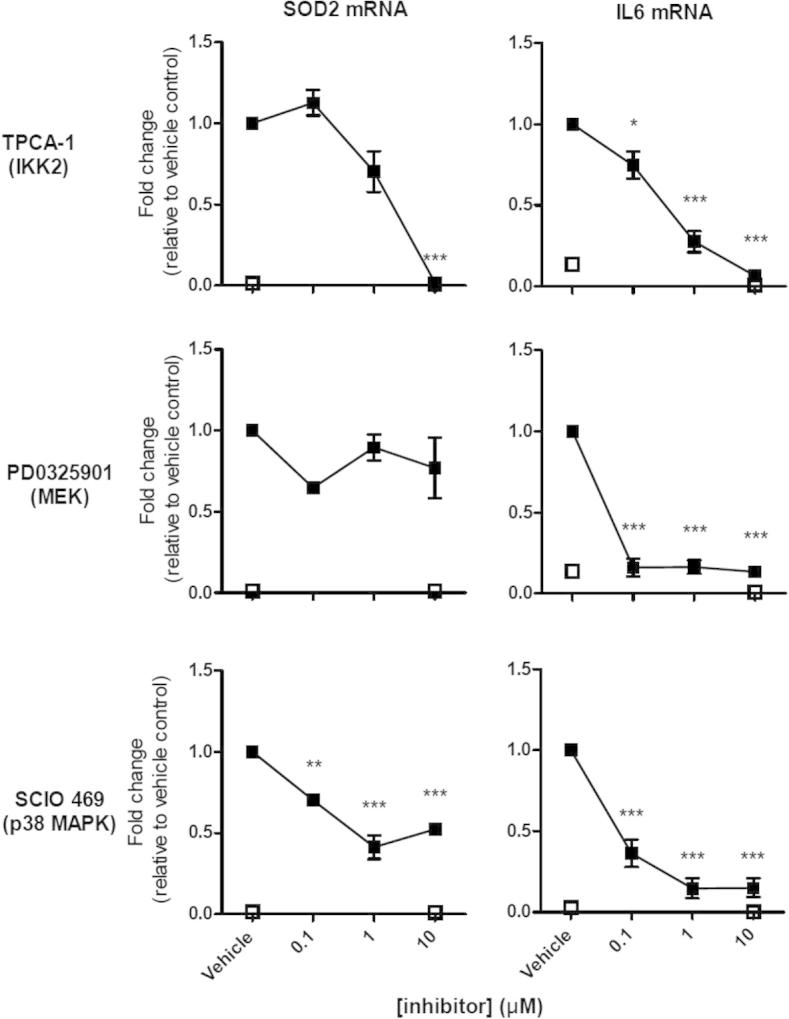
Pharmacological analysis of the signalling pathways that regulate LPS-induced *SOD2* and *IL6* mRNA expression. Human monocytic THP-1 cells were treated with DMSO (vehicle control) or pharmacological inhibitors of IKK2 (TPCA-1), MEK (PD0325901) and p38 (SCIO 469) for 30 min prior to LPS stimulation (4 h). *IL6* and *SOD2* expression was quantified by qRT-PCR. Data is the mean ± S.E.M. of 3 independent experiments. Statistical significance was determined using a one way ANOVA with a Dunnett’s post-test, where ^∗^*P* < 0.05, ^∗∗^*P* < 0.01 and ^∗∗∗^*P* < 0.001.

**Fig. 6:**
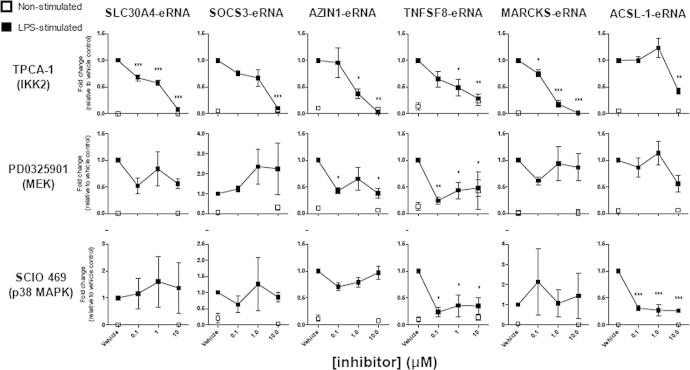
Pharmacological analysis of the signalling pathways that regulate LPS-induced eRNA expression. Human monocytic THP-1 cells were treated with DMSO (vehicle control) or pharmacological inhibitors of IKK2 (TPCA-1), MEK (PD0325901) and p38 (SCIO 469) for 30 min prior to LPS stimulation (4 h). *SLC30A4-eRNA, SOCS3-eRNA, AZIN1-eRNA, TNFSF8-eRNA, MARCKS-eRNA* and *ACSL1-eRNA* expression was quantified by qRT-PCR. Data is the mean ± S.E.M. of 3 independent experiments. Statistical significance was determined using a one way ANOVA with a Dunnett’s post-test, where ^∗^P < 0.05, ^∗∗^P < 0.01 and ^∗∗∗^P < 0.001.

The role of IKK2 upon the LPS-induced response at 4 h was initially examined using the pharmacological inhibitor, TPCA-1. As expected, the LPS-induced *SOD2* and *IL6* expression was repressed with IC_50_ values of 2.7 μM and 0.27 μM, respectively (Fig. 5). Interestingly, all of the LPS-induced eRNAs were significantly repressed following inhibition of IKK2, with IC_50_ values ranging from 0.083 μM (*TNFSF8-eRNA)* to 3.24 μM (*SOCS3-eRNA*) (Fig. 6). It was not possible to determine an IC_50_ value for *ACSL1-eRNA*, as only the highest concentration of TPCA-1 inhibited its expression.

**Fig. 7:**
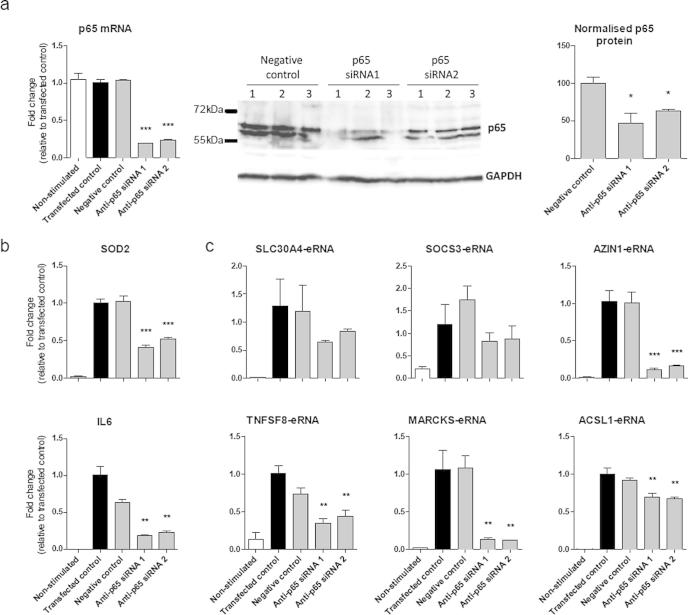
Effect of siRNA-mediated knockdown of p65 NF-κB upon LPS-induced mRNA and eRNA expression. Human monocytic THP-1 cells were transfected with either a non-targeting negative control siRNA or 2 siRNAs targeting p65, for 6 h at 30 nM and then diluted to 10 nM for 42 h, before exposure to buffer or 1 μg/ml LPS for 2 h. The expression of the following was subsequently examined; (a) *p65* mRNA by qRT-PCR and p65 protein by Western blot, (b) *SOD2* and *IL6* mRNA by qRT-PCR and (c) *SLC30A4-eRNA, SOCS3-eRNA, AZIN1-eRNA, TNFSF8-eRNA, MARCKS-eRNA* and *ACSL1-eRNA* by qRT-PCR. Data is the mean ± S.E.M. of 3 independent experiments. Statistical significance was determined using a one way ANOVA with a Dunnett’s post-test, where ^∗^*P* < 0.05, ^∗∗^*P* < 0.01 and ^∗∗∗^*P* < 0.001.

**Fig. 8:**
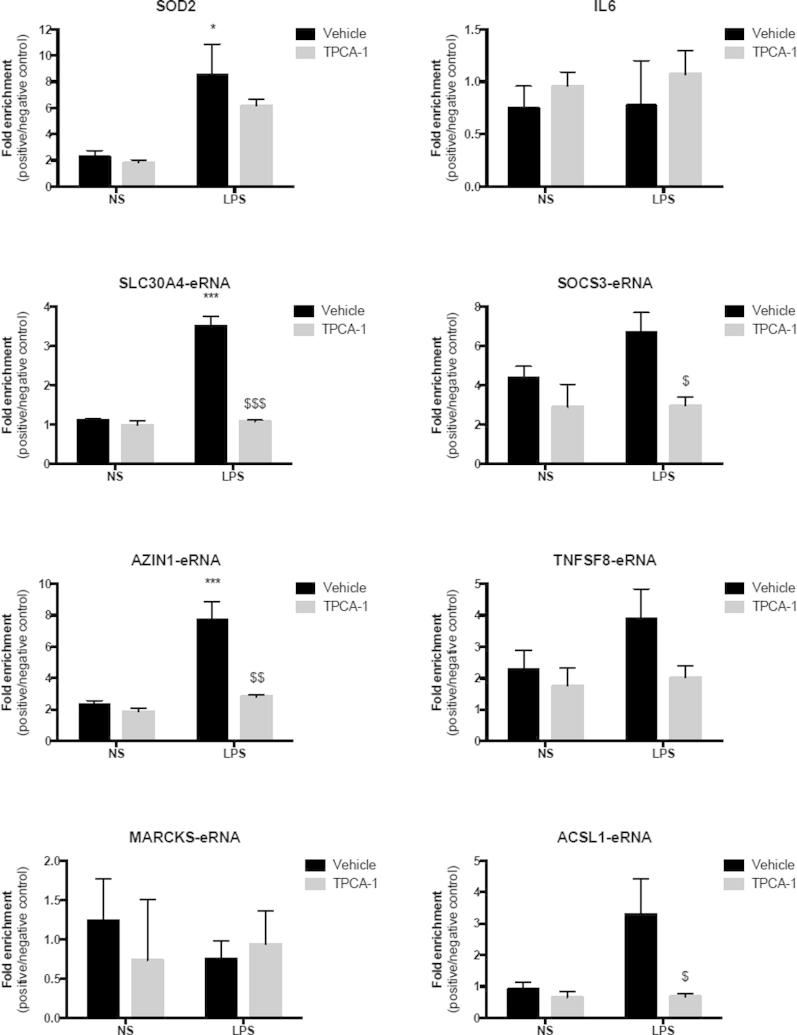
Measurement of NF-κB binding at mRNA and eRNA promoters using chromatin immunoprecipitation. Human monocytic THP-1 cells were exposure to buffer or 1 μg/ml LPS for 1 h in the presence or absence of 10 μM TPCA-1 prior to p65 NF-κB chromatin immunoprecipitation. Binding within the promoter regions of the specified mRNA and eRNA were quantified using qRT-PCR. Data is the mean ± S.E.M. of 3 independent experiments. Statistical significance was determined using a one way ANOVA with a Dunnett’s post-test, where ^∗^*P* < 0.05 and ^∗∗∗^*P* < 0.001 versus non-stimulated controls and ^$^*P* < 0.05 and ^$$$^*P* < 0.001 versus stimulated samples in the absence of TPCA-1.

To provide additional evidence of a role for NF-κB in LPS-induced eRNA expression we undertook studies using siRNA-mediated knockdown and chromatin immunoprecipitation (ChIP) in combination with qRT-PCR. Using two siRNAs targeted to the p65 subunit of NF-κB we showed a significant decrease in the mRNA expression and protein level of p65, whereas no knockdown was observed following transfection with the negative control (Fig. 7a). Knockdown of p65 by both siRNAs significantly reduced the LPS-induced mRNA levels of *SOD2* and *IL6* (Fig. 7b). Similarly, the LPS-induced expression of all of the eRNAs was also impaired. Thus, significant decreases in LPS-induced expression of *AZIN1-eRNA, TNFSF8-eRNA, MARKS-eRNA* and *ACSL1-eRNA* were observed although the decreases in LPS-induced expression of *SLC30A4-eRNA* and *SOC3-eRNA* did not reach significance (Fig. 7c). Chromatin immunoprecipitation (ChIP) using an antibody to the p65 RelA DNA binding, was employed to measure NF-κB activation and binding in the absence and presence of IKK2 inhibitor. Although this only reached significance in 50% of the genes examined, these studies showed a general trend for increased NF-κB binding within the predicted promoter regions of *SOD2*, *SLC30A4-eRNA*, *AZIN-1-eRNA*, *SOCS3-eRNA*, *TNFSF8-eRNA* and *ACSL1-eRNA* and that this was reversed in the presence of the IKK2 inhibitor (Fig. 8). In contrast, we observed no NF-κB binding in the promoter region of *IL6* or *MARCKS-eRNA* (Fig. 8). The reasons for the variability in the ChIP studies is unclear although it could relate to a number of factors including the difficulties in predicting the NF-κB binding region (for qRT-PCR probe design) and the transient nature of NF-κB binding. Overall, this data suggested that the NF-κB signalling pathway has a key role in regulating LPS-induced eRNA expression.

### 3.6 LPS-induced eRNA expression is regulated by the MAP kinase signalling pathways

**Fig. 9:**
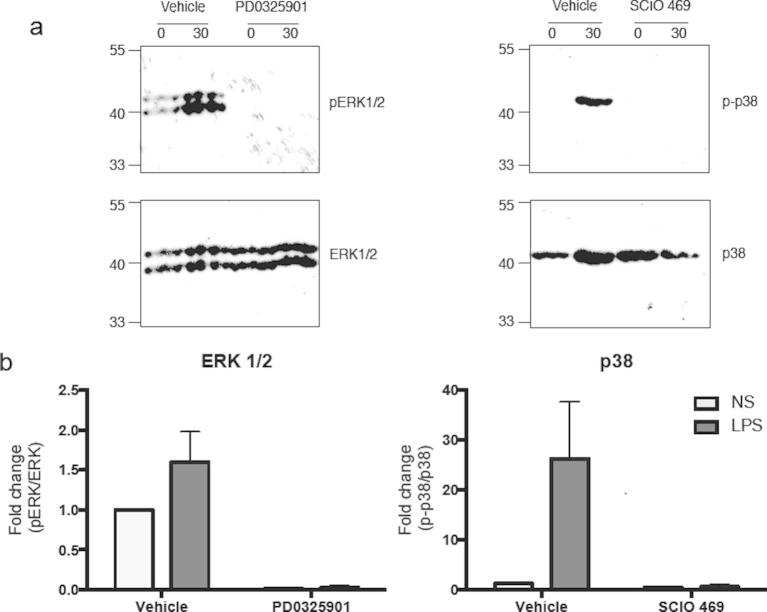
Inhibition of LPS-mediated ERK-1/2 and p38 phosphorylation. Human monocytic THP-1 cells were treated with DMSO (vehicle) or 10 μM of pharmacological inhibitors of MEK (PD0325901) or p38 MAPK (SCIO 469) for 30 min prior to LPS stimulation (30 min). (a) The level of ERK-1/2 and p38 phosphorylation was assessed by Western blot (representative experiment shown) and (b) the levels of phosphorylation were quantified using ImageJ software. Data is the mean ± S.E.M. of 3 independent experiments.

Using Western blotting, we were able to demonstrate activation of the ERK-1/2 and p38 using phosphoantibodies to detect the activation forms of these MAP kinases (Fig. 9). Importantly, following the observation that this phosphorylation/activation could be completed attenuated in the presence of the selective MEK-1/2 (PD0325901) and p38 (SCIO 469) inhibitors (Fig. 9), these compounds were employed to examine the role of these pathways in the regulation of eRNA expression. In addition to *IL6*, these studies indicated that the expression of *AZIN1-eRNA* and *TNFSF8-eRNA* were mediated via the ERK-1/2 pathways (Figs. 5 and 6). In the case of p38 inhibitor, our investigations showed a role for this pathway in the LPS-induced expression of *SOD2*, *IL6*, *TNFSF8-eRNA* and *ACSL1-eRNA* (Figs. 5 and 6). Of relevance, we were unable to demonstrate phosphorylation/activation of JNK-1/2 or an effect of the JNK inhibitors SP600125 (at 10 μM) upon LPS-induced expression of *SOD2*, *IL6* and *eRNAs* (data not shown). It therefore appears that in addition to NF-κB, the expression of a number of eRNAs are regulated via ERK-1/2 and p38.

### 3.7 Discussion and conclusions

Advances in sequencing technology have begun to reveal the startling complexity of the human epigenome including the widespread occurrence of enhancer regions, with approximately 400 000 putative enhancers having been identified as part of the ENCODE project [23]. Importantly, it is becoming apparent that the action of these enhancers is dependent upon the production of eRNAs, an observation supported by the FANTOM project that identified 43 000 bi-directionally transcribed eRNAs across a diverse range of human tissues [16]. Interestingly, dynamic activation and repression of enhancers has been shown to occur upon exposure of immune cells to inflammatory stimuli, suggesting that enhancers have a key role in regulating inflammatory gene expression [13,14,24]. Furthermore, one of the key findings of the FANTOM project was that immune cells such as monocytes, in contrast to terminally differentiated cell types such as epithelial cells, express high relative numbers of eRNAs [16].

However, despite the increasing evidence to suggest the importance of eRNAs in the immune response, little is known about the signalling pathways that regulate their expression. To address this question, we have characterised the mechanisms that regulate 6 eRNAs that are induced in response to LPS [13]. All of these eRNAs overlapped with enhancer regions identified by the FANTOM project [16] and contained numerous binding sites for inflammatory transcription factors. In support of the observation that eRNAs regulate gene expression [8,14], we showed rapid induction of eRNA expression that preceded or correlated with expression of other inflammatory genes upon LPS exposure. Furthermore, distribution studies showed that the majority were located in the nucleus and that their actions might therefore be mediated in *cis*. Interestingly, the expression of two of the examined eRNAs was not restricted to the nucleus, suggesting that some eRNAs might act in the cytoplasm. Examination of the intracellular signalling pathways indicate that the NF-κB signalling pathway has a central role in regulating LPS-induced eRNA activation, consistent with the reports that NF-κB is required for activation of enhancers in inflammation [14,25]. However, the expression of individual eRNAs were also dependent on ERK-1/2 and p38, implying that transcription and potentially the activation of these enhancers requires multiple transcription factors in addition to NF-κB.

In moving forward it will be necessary to investigate the function of these immune related eRNAs and the mechanisms that regulate their expression. On a genome scale, our previous studies have shown that eRNAs expression is correlated with the localised expression of mRNAs. Interestingly, the expression of the nearest mRNAs which includes *SLC30A4 (2 fold)*, *AZIN1 (2 fold)*, *SOC3 (14 fold)*, *TNFSF8 (3 fold)*, *MARCKS (7 fold)* and *ACSL1 (8 fold)*, were all shown to be induced following LPS exposure. This correlation implies that these eRNAs indeed function to regulate localised gene expression although this would need to be confirmed by eRNA knockdown. Possibly as a result of their nuclear localisation and rapid induction, these eRNAs knockdown studies have proven problematic. However, this situation could be transformed by the recent developments in the area of CRISPRi, which permits inhibition of eRNA transcription rather than the traditional post-transcription approaches involving siRNAs and antisense [26]. Clearly, the other issue is the exact mechanism by which eRNA expression is regulated within the enhancer regions. In addition to NF-κB, this report indicates that transcription factors downstream of ERK-1/2 and p38 such as AP-1 might be also involved in eRNA transcription. As with our studies on p65 (NF-kB), this question can be addressed using ChIP to assess AP-1 binding at eRNA sites and examining the effect of AP-1 knockdown upon eRNA and mRNA expression.

In conclusion, we have characterised the mechanism that regulate the expression of multiple immune-regulated eRNAs and shown that like inflammatory gene expression, this occurs through divergent signalling pathways.

## Figures and Tables

**Table 1:** Sequences of qRT-PCR primers and siRNAs.

Primers		
qRT-PCR	Forward primer	Reverse primer
*18S rRNA*	AAACGGCTACCACATCCAAG	CCTCCAATGGATCCTCGTTA
*IL6*	ACTCACCTCTTCAGAACGAATTG	CCATCTTTGGAAGGTTCAGGTTG
*SOD2*	GCTCCGGTTTTGGGGTATCTG	GCGTTGATGTGAGGTTCCAG
*p65*	ATGTGGAGATCATTGAGCAGC	CCTGGTCCTGTGTAGCCATT
*MALAT1*	CTTAAGCGCAGCGCCATTTT	GGCCAGCCTATAAGGACAGC
*MT-CYB*	TAGACAGTCCCACCCTCACA	CGTGCAAGAATAGGAGGTGGA
*MARCKS-eRNA*	CACCACCCAGAAGATGCTGAA	GGCCATGCGTTATGTTTCTTGA
*ACSL1-eRNA*	ACCATCGCAAGCTGCCCAGG	CCTGGGTCCGCCCGTAGACAT
*AZIN1-eRNA*	GGGGTGTGTGGGTGATTAGG	CCCTGGAGTACCAAGCTTCC
*TNFSF8-eRNA*	TCCTCCTCCTAAGTGTGGGT	CCACACAGGAGGGATGCAAG
*SLC30A4-eRNA*	GTGATGTGACAGCGTACGGCA	ACTTCCTTTGGGTTTGGGGCAGA
*SOCS3-eRNA*	TGTGGGTGGAGACACCTGGGC	CTGGCTTCTCTCAGCCGGGG

ChIP	Forward primer	Reverse primer

*SOD2*	CCTGGTGTCAGATGTTGCCT	GGGAAAAGGCCCCGTGATTT
*IL6*	AAAGGAGTCACACACTCCACC	CCTGTGAGCGGCTGTTGTA
*SLC30A4-eRNA*	TTTTGCGCCTTCCTGGTGTT	GGACTCTTGCTCTCACCAGC
*SOCS3-eRNA*	TCTGCAAACCTGGTTGGTCC	GATCCCTGGCGTGCCTATTC
*AZIN1-eRNA*	AGGTTAGCTGGTGTTTTCTTATTGG	GGCAACCCTACCACTTTAGGT
*TNFSF8-eRNA*	GATGACACTTGAGTCCGCCA	AGCAGGGGAACGGCAATTAT
*MARCKS-eRNA*	CACCACCCAGAAGATGCTGAA	GGCCATGCGTTATGTTTCTTGA
*ACSL1-eRNA*	GGGAAAGGGACATACCTGGC	CCTCTCAGTCACAAGACGGC
		
siRNAs		

siRNA name	Sequence	Source

Scrambled	UGGUUUACAUGUCGACUAA	Dharmacon
Anti-p65 1	GGAUUGAGGAGAAACGUAA	Dharmacon
Anti-p65 2	CCCACGAGCUUGUAGGAAA	Dharmacon

**Table 2:** Transcription factor binding sites, identified in the ENCODE project [23], and transcribed enhancer regions identified by the FANTOM project that overlap with eRNA gene bodies. Overlap with eRNA promoters (from +500 bp to −2 kb relative to the transcription start site) is indicated in brackets.

Gene	p65	JUN	FOS	JUND	FANTOM enhancer
*ACSL1 eRNA*	5 (0)	2 (0)	7 (0)	4 (1)	3 (0)
*MARCKS eRNA*	5 (0)	1 (0)	4 (0)	4 (0)	7 (0)
*AZIN1 eRNA*	1 (0)	0 (0)	1 (0)	0 (0)	2 (0)
*TNFSF8 eRNA*	3 (1)	2 (0)	3 (1)	3 (0)	4 (1)
*SLC30A4 eRNA*	1 (0)	1 (0)	4 (1)	3 (0)	4 (0)
*SOCS3 eRNA*	3 (1)	0 (1)	1 (1)	1 (2)	1 (1)
*IL6*	0 (1)	2 (3)	1 (1)	2 (0)	0 (0)
*SOD2*	2 (1)	0 (1)	1 (2)	2 (3)	0 (0)
